# Spontaneous Spinal Epidural Hematoma from Rivaroxaban

**DOI:** 10.5811/cpcem.2018.2.37096

**Published:** 2018-04-05

**Authors:** Charlotte Goldfine, Catherine Glazer, Richard M. Ratzan

**Affiliations:** University of Connecticut School of Medicine, Hartford Hospital, Department of Emergency Medicine, Hartford, Connecticut

## Abstract

Spontaneous spinal epidural hematoma (SSEH) is a rare diagnosis. One known risk factor is anti-coagulation medication. We present a case of SSEH in a 74-year-old male on rivaroxaban therapy who clinically presented with an intermittently resolving and then worsening neurological exam. Due to the extremely high morbidity and mortality associated with this diagnosis, it is important to be aware of the various presentations and adverse effects related to novel anticoagulation.

## INTRODUCTION

Spinal epidural hematomas are a rare event. The estimated incidence is 0.1 per 100,000 per year with a frequency accounting for less than 1% of spinal epidural space-occupying lesions.^1^ Spinal epidural hematomas are classified based on their etiology, with trauma being the most common cause.^2^ Other etiologies that have been described include invasive spinal procedures such as lumbar puncture and myelography, arteriovenous malformations, bleeding disorders, pregnancy, and spinal manipulation.^3^,^4^

Even more rarely, spinal epidural hematomas can occur spontaneously. Cases have been reported with coughing, stretching, and even playing the trombone.^4^–^6^ In most cases (40–60%) the cause is idiopathic, with no clear inciting factor or underlying etiology.^7^ When a cause can be found it tends to be related to a minimal amount of trauma or physical effort that would not be expected to cause significant bleeding.^8^ Most cases occur in the fourth or fifth decade of life.^9^ There have also been reports of spontaneous spinal epidural hematomas (SSEH) occurring in pediatric patients in whom the disease can be more difficult to diagnose and more neurologically devastating.^10^

There is no clear consensus on whether the pathophysiology of the hemorrhage is arterial or venous.^11^ Several risk factors leading to SSEH have been identified. 12 One of the leading risk factors is the use of anticoagulation medications, second only to idiopathic causes.^13^ The most commonly associated anticoagulant medication resulting in SSEH is warfarin.^13^ There have only been four previously documented cases of SSEH on Xa inhibitors.^14^–^17^

Spinal epidural hematomas typically present clinically as a cord compression syndrome. The most common presenting complaint is the acute onset of neck or back pain with associated neurological deficit such as weakness of the extremities.^3^, ^18^ The symptoms vary depending on the location of the hematoma, resulting in a variety of presentations.^3^ There have been reports of SSEH mimicking stroke and acute coronary syndrome.^1^,^19^ In most cases, the symptoms persist until the hematoma is surgically evacuated, although spontaneous resolution – completely or partially - is not rare.^20^ In our patient the neurological symptoms transiently improved and then worsened again throughout his course. The best imaging modality to appropriately identify SSEH is magnetic resonance imaging (MRI). If not appropriately diagnosed and treated, the cord compression can lead to permanent disability such as persistent weakness and even paraplegia. Early treatment is associated with improved outcomes, and therefore it is an important syndrome to recognize in emergency medicine.^19^

## CASE REPORT

A 74-year-old man on rivaroxaban for paroxysmal atrial fibrillation presented with the gradual onset of neck pain. The pain started while he was walking in the woods and was associated with progressive weakness and numbness from the clavicles downward. He was able to walk back to his cabin, but upon arrival he was unable to maintain his balance and fell forward striking his head. He denied any loss of consciousness. He was unable to move for about 45 minutes. Emergency medical services was called and they noted the patient to be completely paralyzed with fecal incontinence. He also had shortness of breath. During his transport to the emergency department (ED) he started to regain strength and feeling in his arms and legs and improvement of his shortness of breath. He denied any recent fevers, upper respiratory infection, rash or tick bites. All other review of systems was negative. Past medical history was significant for hypertension, atrial fibrillation, and coronary artery disease.

On initial examination, the patient was alert and oriented to person, place, and time. Temperature was 92.0 degrees Fahrenheit tympanic, pulse 56 beats per minute, respiratory rate 20 breaths per minute, blood pressure 122/56 mmHg, oxygen saturation 100% on four liters nasal cannula. Lungs were clear to auscultation bilaterally without wheezes, rhonchi, or rales. Heart was regular rate and rhythm, without any murmurs. Abdomen was soft, non-tender, non-distended with normal bowel sounds. Back had no evidence of trauma or deformity, non-tender to palpation. On extremity exam he had 4/5 strength in all extremities. After 20 minutes in the ED, he developed flaccid paralysis of all extremities with 0/5 strength. His voice also became softer and he developed shortness of breath. After five minutes he was able to wiggle his left toes. Over the next hour he slowly regained strength in his right lower extremity and his voice returned to normal. After another 40 minutes the patient was able to move his right upper and lower extremity and wiggle his toes on his left lower extremity. He was still unable to move his left upper extremity.

A computed tomography angiography (CTA) of the chest, abdomen, and pelvis was performed and did not show any evidence of aortic dissection. MRI of the cervical spine showed a prominent epidural hematoma, primarily dorsal, that extended from the foramen magnum down to cervical vertebra C7 and contributed to severe mass effect on the thecal sac (Image). Findings were the worst at C2–C3, where the hematoma measured 7–8 mm in width with compression of the cervical spinal cord and associated cord signal abnormality. In retrospect, findings were also visible on the preceding CTA. Patient was taken emergently to surgery for evacuation of the hematoma.

## DISCUSSION

There have only been four other cases of SSEH formation while on rivaroxaban^14^–^17^ with an additional two cases of SSEH formation.^21^, ^22^ Rivaroxaban is an oral anticoagulant medication that works as a direct factor Xa inhibitor.^23^ Oral anticoagulation medications are increasingly being used for stroke prevention in non-valvular atrial fibrillation and deep vein thrombosis, as they overcome some of the difficulties when using the older standard medications that are more complex to administer or require closer monitoring.^23^ There is no specific antidote for rivaroxaban; therefore, nonspecific reversal agents such as fresh frozen plasma or a four-factor prothrombin complex concentrate are used in severe, life-threatening cases where reversal is necessary.^24^, ^25^

**CPC-EM Capsule**

What do we already know about this clinical entity?

Spontaneous spinal epidural hematoma (SSEH) is a rare diagnosis. There are several risk factors, including anticoagulation medications. Typically the disease presents as a cord compression syndrome.

What makes this presentation of disease reportable?

This case was interesting in that the neurological symptoms were waxing and waning. There have also been few cases reported of SSEH on rivaroxaban.

What is the major learning point?

While SSEH will usually present as a cord compression syndrome, it is important to consider this diagnosis in patients on rivaroxaban or other novel anticoagulation medications with an atypical presentation.

How might this improve emergency medicine practice?

Due to the extremely high morbidity and mortality associated with this diagnosis, it is important to be aware of the various presentations and adverse effects related to novel anticoagulation.

This case was interesting in that the neurological exam was waxing and waning, rather than a clear neurological deficit as one would expect from a compression syndrome. In other cases of SSEH caused by novel anticoagulants, two resolved spontaneously without the need for surgery, whereas the third and fourth required surgical intervention.^14^–^17^

As the use of new anti-coagulant medications such as rivaroxaban increases, there will likely be an increase in adverse events and new complications. SSEH may be one of them. The varied presentations and lack of clear inciting factor make SSEHs difficult to diagnose in the ED. While this is a rare diagnosis, anticoagulation medication is a known risk factor. Given the increased morbidity and mortality associated with this diagnosis and the use of new anticoagulation medications, it is important to be aware of these unusual presentations of SSEHS.

## CONCLUSION

Spontaneous spinal epidural hematomas are a rare but serious diagnosis. Knowing the risk factors associated with this serious illness is important to recognize in emergency medicine. As new anticoagulant medications such as rivaroxaban are becoming more widely used, adverse effects from these medications will likely become more prevalent, and SSEH is likely one of them.

Documented patient informed consent and/or Institutional Review Board approval has been obtained and filed for publication of this case report.

## Figures and Tables

**Image:**
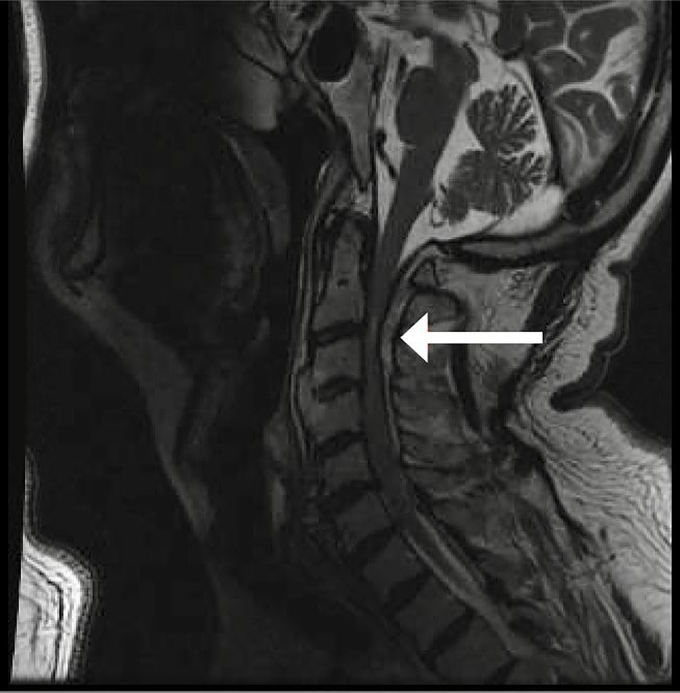
Magnetic resonance imaging (T2 weighted) in sagittal view demonstrating spontaneous spinal epidural hematoma from the foramen magnum to cervical spine level 7 as indicated by the arrow.
